# Recent Trends in Surgical Approach to Thyroid Cancer

**DOI:** 10.3389/fendo.2021.699805

**Published:** 2021-06-02

**Authors:** Leonardo Rossi, Gabriele Materazzi, Sohail Bakkar, Paolo Miccoli

**Affiliations:** ^1^ Department of Surgical, Medical, Molecular Pathology and Critical Area, University of Pisa, Pisa, Italy; ^2^ Department of Surgery, Faculty of Medicine, Hashemite University, Zarqa, Jordan

**Keywords:** thyroid cancer, RATT, MIVAT, TOETVA, minimally invasive

## Abstract

Over the past decade, the incidence of thyroid cancer has rapidly increased worldwide, and thyroid surgery has become one of the most common performed surgical procedure. Even though conventional open thyroidectomy remains the gold standard, this approach leaves a neck scar which could be worrying mainly for young women. The recent progress in surgical technology, as well as patient cosmetic requests, have led to the development of alternative access to the thyroid lodge. Thus, alternative techniques have been established in order to potentially provide a more appealing cosmetic result, both with a minimally-invasive cervical or remote-access approach. However, the introduction of these new techniques was initially approached with caution due to technical challenges, the introduction of new complications and, above all, skepticism about the oncologic effectiveness. Among several alternative approaches proposed, the minimally invasive video-assisted thyroidectomy and the robot-assisted transaxillary thyroidectomy became popular and obtained the favor of the scientific community. Moreover, the recent introduction of the trans-oral endoscopic thyroidectomy with vestibular approach, although the safety and the efficacy are still under discussion, deserves particular attention since it represents the only technique truly scarless and provides the best cometic result. The purpose of this article is to provide an overview of the current main alternative approaches for the treatment of thyroid cancer with particular focus on the oncological effectiveness of the procedures.

## Introduction

The incidence of thyroid cancer has markedly increased worldwide during the last decade, with thyroid surgery becoming one of the most common surgical procedures mainly due to an increasing use of neck ultrasonography and fine needle aspiration biopsy (FNAB). The exponential growth of these procedures in the last years have enabled to detect many more cases of tumors than in the past, and in particular more microcarcinomas. Although health screening programs leading to earlier detection surely play a role in the increasing identification of thyroid tumors, other environmental factors also may contribute (1).

The conventional open approach to thyroidectomy, initially proposed by Theodore Kocher in the late 1800s, leaves a neck scar that is associated with great concern, in particular for young women, who are very sensitive to cosmesis. This issue led to the introduction in the late 90s of alternative approaches to obtain a better cosmetic result or even avoid a visible neck scar (2–5).

Minimally invasive video-assisted thyroidectomy (MIVAT), which was originally described by Miccoli et al. (2) in the late 1990s, has proved to be a safe procedure that harbors potential advantages in cosmetic results and postoperative outcomes compared with the conventional procedure, including shorter scar length, better cosmesis, and reduced pain. Although descriptions of this procedure date back to more than 20 years, it still remains one of the favorite endoscopic techniques to remove the thyroid gland (6, 7).

Furthermore, during the past decade, an imposing number of different remote‐access approaches have been described as a method of removing the thyroid gland while avoiding a neck scar. These techniques have been developed to potentially provide a cosmetically more appealing result for some patients and have often resulted as an expression of different habits and expectations of patients of different geographic regions and cultures. However, although initially surrounded by skepticism due to technical challenges, the introduction of new complications, and concerns about oncologic safety and cost, some of them have been approached progressively more widely by the community of endocrine surgeons. Anyhow, it is strictly recommended to adhere to selection criteria and to consider that these techniques should be approached by surgeons performing a high volume of thyroid surgery (8).

This review provides an overall evaluation of the main alternative approaches to conventional thyroidectomy (CT), such as minimally-invasive video-assisted thyroidectomy (MIVAT), robot-assisted trans-axillary thyroidectomy (RATT), and the transoral endoscopic thyroidectomy with vestibular approach (TOETVA), for the treatment of thyroid cancer, focusing on the oncologic safety and effectiveness of these procedures for this cohort of patients.

## Minimally Invasive Video-Assisted Thyroidectomy (MIVAT)

Since its introduction in the late 1990s, MIVAT has been worldwide adopted thanks to its reproducibility and its comparable outcomes to the conventional open approach. This minimally invasive video-assisted technique permits surgeons to safely perform thyroidectomy and provides the benefits of the typical advantages proper of endoscopic surgery, including magnified vision, better cosmetic results, and reduced postoperative pain (9, 10).

Although initially introduced into clinical practice for the treatment of small benign thyroid nodules (2), the use of MIVAT for the treatment of thyroid cancer gained progressively more popularity, and several case series demonstrated its feasibility and safety even in this cohort of patients (11–13). All in all, ideal candidates for MIVAT are patients with an ultrasound-estimated thyroid volume not exceeding 25 ml with nodules smaller than 35 mm. On the other hand, absolute contraindications for MIVAT are large multinodular goiters, previous neck surgery or irradiation, locally invasive carcinoma, presence of lateral neck compartment lymph node metastasis. The presence of enlarged lymph nodes in the central compartment of the neck is not necessarily a contraindication since MIVAT proved to be fit even for VIth level lymphadenectomy, although we believe that it should be performed with caution and only in cases of incidentally intraoperative discovery of enlarged lymph nodes (14). Moreover, caution must be taken in case of small thyroid cancers when located very posteriorly because they could have an extracapsular infiltration: this situation could represent a reason for a prompt conversion to CT (7). Further, presence of thyroiditis, adverse anatomical aspects (short neck in obese patients) and hypervascularization of the thyroid gland, represent relative contraindications to MIVAT (14).

We investigated the oncologic completeness of MIVAT compared with CT in a prospective study of 33 patients: 16 underwent MIVAT and 17 underwent nearly total CT. No statistically significant differences were found in I-131 uptake and serum thyroglobulin levels, showing that the completeness obtained by MIVAT was comparable to the one by CT (15).

Furthermore, the excellent oncologic outcomes of this minimally invasive approach were confirmed in another study involving patients affected by differentiated thyroid carcinoma (DTC) with a median follow-up of 5 years (16). The study enrolled 221 patients: 171 underwent MIVAT and 50 underwent CT. At the time of remnant ablation, no differences in serum thyroglobulin, thyroid stimulating hormone (TSH) levels, or I-131 neck uptake were observed between the two groups. After a 5-year follow-up, the two groups were comparable in outcomes, with no thyroid cancer-related death or recurrence documented in either group. Finally, the cumulative dose of I-131 needed to definitively cure the thyroid cancer was the same regardless of the surgical approach, indirectly confirming that the two techniques are superimposable in oncologic completeness (16).

Our entire case series of DTC treated by means of MIVAT was evaluated in 2015 (9). In particular, 528 patients presenting with thyroid cancer were monitored for a median period of 7.5 years. The evaluation of thyroglobulin serum levels showed optimal results, as did the radioactive iodine dose required for completion and for recurrence in this cohort. Relapse was documented in 24 patients (4.5%); of these, 14 were treated surgically, and 10 were treated with a repeat administration of radioactive iodine. In the same period (2000–2009), 234 patients with a comparable stage disease underwent a CT. The cure rate in this control group was very similar: 80% of the patients were cured in the same follow-up interval (9). Although only 7.2% of patients in our case series did not receive I-131, it seems that according to the present standard guidelines, in most, if not all cases, radioactive iodine therapy would not be necessary after a total thyroidectomy performed *via* MIVAT when selection criteria are carefully followed (17).

Moreover, the 15 patients carrying a *RET* gene mutation who underwent a prophylactic thyroidectomy and central neck dissection *via* the MIVAT approach showed undetectable serum levels of calcitonin (9). Other authors have reported similar results (18).

Several institutions have assessed the efficacy of MIVAT in the treatment of thyroid cancer (19, 20). Del Rio et al. (19) performed a prospective study in 2015 to compare the oncologic outcomes in patients with DTC treated with MIVAT versus CT. Of 172 patients who were enrolled, 67 were treated with the minimally invasive technique and 105 with the open approach. After a mean follow-up of 5 years, the authors reported no statistically significant difference in oncologic efficacy; in particular, the two techniques were comparable for disease control after post-ablation scintigraphy and thyroglobulin levels (19). Accordingly, Lai et al. (20) evaluated the oncologic completeness of MIVAT in 16 patients with low- or intermediate-risk thyroid cancer, 6 of whom underwent incidentally (n = 5) or intentionally (n=1) central compartment neck dissection along with thyroidectomy. The radioiodine uptake and the radioiodine dose delivered in patients who underwent MIVAT were comparable to CT, and radioiodine ablation showed undetectable thyroglobulin levels (20).

Notably, Lombardi et al. (21) performed a comparative study with the goal to demonstrate the safety and feasibility of MIVAT and central neck dissection. They reported outcomes for 52 consecutive patients who were treated by means of the minimally invasive technique, and 52 patients who were treated by means of the conventional approach. They concluded that the two techniques were comparable in lymph nodes harvest, serum thyroglobulin off levothyroxine, postoperative ultrasound neck scan, and postoperative radioiodine uptake. The authors claimed that the endoscopic view allows an accurate exploration of the central compartment and enables identification of even slightly enlarged lymph nodes. Other authors also reported comparable oncologic results between MIVAT and CT with associated central neck dissection in patients affected by thyroid carcinoma (11, 22).

On one hand, we believe that video-assisted central neck dissection must be performed with caution and only in patients with intraoperative unexpected discovery of enlarged lymph nodes. On the other hand, this technique is appropriate for prophylactic central neck dissection in patients who are mutated RET carriers (9, 18).

Regarding complications, several studies dealing with MIVAT reported data comparable to CT, strengthening the idea that this is a safe technique (9). Indeed, although the narrow space and the few degrees of freedom, these outcomes can be achieved thanks to the magnified vision of the endoscope which allows an easy identification of parathyroid glands and recurrent laryngeal nerves (7).

The indications for MIVAT have been extended over the years from small benign nodules to low- and intermediate-risk thyroid cancer, showing a level of oncologic safety comparable to CT. Moreover, reduced pain and hospital stay, and increased patient satisfaction are the strengths of this approach. After a long debate, the initial reluctance has been swept away, and this technique has gained acceptance worldwide in the treatment of selected thyroid carcinoma. To obtain excellent results, strict adherence to criteria selection is required, especially at the beginning of the experience, and it is strongly recommended to perform MIVAT in high-volume centers by trained endoscopic endocrine surgeons. Unfortunately, MIVAT is a technique limited to a niche of patients due to the volume of the gland and the size of the nodule required to fit the selection criteria. Depending on the geographic area, only approximately 20% to 30% of patients may benefit of this approach (20).

## Robot-Assisted Transaxillary Thyroidectomy (RATT)

The desire to avoid neck scarring after thyroid surgery has resulted in the development of endoscopic and robotic remote access techniques. The gasless transaxillary endoscopic thyroidectomy was proposed in November 2001 in South Korea at the Yonsei University College of Medicine, Seoul, to satisfy this necessity (23). However, endoscopic thyroidectomy showed several limitations, such as difficulty in instrument handling and restricted vision (24). The introduction of surgical robots was thought to overcome drawbacks of endoscopic surgery and to provide technical improvements, including magnified 3-dimensional (3D) vision, tremor-filtering systems, and additional degrees of freedom (25).

The approval of the da Vinci surgical system (Intuitive Surgical, Sunnyvale, CA, USA) by the United States Food and Drug Administration in 2000 made its use progressively more widespread (25). An important turning point in the development of the robot-assisted transaxillary approach was the description of the procedure using a single access that avoided the accessory sternal incision (26, 27). This less invasive procedure provides better cosmetic outcomes and improves patient comfort, arousing interest among the medical community (26).

The robot-assisted transaxillary thyroidectomy (RATT), popularized by Chung et al, who published their experience with 5000 cases in 2018, became widely used in countries in East Asia, although still under discussion in Europe and the Americas (25, 28). The negative connotation of having a horizontal neck scar, which is thought to denote death in Asian culture, may have played a role in the rapid spreading of this technique (29). Differences in body mass index and anthropometric characteristics and greater size of goiters and cancers, combined with the elevated costs of the procedure and the need of training, have hindered the diffusion of this approach in the United States and Europe (28). To date, RATT, although excellent results in feasibility, safety, and patient satisfaction are described, is limited to play a niche role in selected patients with appropriate pathology in high-volume centers (28).

Overall, indications for RATT varies among the centers, but nowadays substantially both benign pathologies and well-differentiated low risk thyroid carcinoma can be approached with this technique. Guidelines recommended to limit RATT to patients affected by well-circumscribed nodule < 3 cm and with thyroid lobe < 5–6 cm in the largest dimension (8). Moreover, previous neck or breast surgery are usually considered contraindications, as well as neck radiotherapy, pacemaker implant, shoulder arthrosis, previous shoulder surgery, substernal extension and Grave’s disease. Nonetheless, indications were progressively expanded as the experience increase and some Institutions performed RATT even in more advanced cases (25).

With regard of complications, several studies reported comparable outcomes between RATT and CT (30, 31). In particular, no statistically significant differences were reported in terms of classic complications (hypoparathyroidism and RLN palsy) rate (31). Moreover, the introduction of potential new complications, which was seen with great concern at the beginning of the experience, deserves a special mention. This group of unconventional complications includes: brachial plexus injury, axillary flap perforation, tracheal injury and surgical-track recurrence. Although patients should be informed of these additional risks, fortunately these complications are extremely rare and, concerning brachial plexus injury, almost always transient. Indeed, its incidence is reported up to 0.2% of patients, but it resulted permanent in 0.04% (32).

Several systematic reviews and meta-analysis reported oncologic outcomes that are equivalent to those of conventional thyroidectomy in terms of completeness and recurrence rate (31, 33).

Lee and colleagues (34) comparatively studied 94 patients who had undergone total thyroidectomy with central neck dissection. The patients were divided between those who underwent robotic (n = 43) and conventional (n = 51) approaches. The authors reported a similar number of retrieved lymph nodes, and no significant differences between the two groups were documented in stimulated thyroglobulin levels acquired during whole-body scans. Moreover, the ablation success rate was similar between the two approaches, and the follow-up ultrasound examination documented no abnormal findings in either group (34). The same authors, in a long-term follow-up evaluation, reported comparable outcomes between CT and RATT in anti-thyroglobulin antibodies, serum thyroglobulin, locoregional recurrence rate, and disease-free survival, claiming that RATT has superimposable impact to CT regarding oncologic completeness (35).

Once reliability of RATT for DTC was ascertained and the surgical skills increased over the time, indications progressively expanded to include more aggressive diseases. In 2018, Chung and colleagues (25) reported their experience with 4804 patients with thyroid cancer. First, it is worth mentioning that almost two-thirds of all of the operations consisted in less-than-total thyroidectomy and only one-third in bilateral total thyroidectomy. Moreover, this cohort of patients presented a medium tumor size of only 0.8 ± 0.6 cm. Anyway, as robotic experience increased, the authors were able to successfully treat even advanced cases, such as those with adjacent muscles invasion or perinodal infiltration. In particular, the authors enrolled 25 patients at T4a stage, and successful preservation of the invaded organs was obtained in 20 patients (25).

Concerning N stage, central neck lymph node metastases and lateral neck lymph node metastases were found in 1407 patients (29.3%) and 363 patients (7.6%), respectively, with a mean number of retrieved central and lateral lymph nodes of 6.3 ± 5.1 and 34.1 ± 17.5, respectively (25). The fine dissection allowed by the robotic system, along with the magnified 3D vision, enables an accurate lymph nodes removal with the number of harvest lymph nodes comparable to the number obtained with open surgery (28). Lee et al. (36) accordingly reported that RATT with modified radical neck dissection provided similar oncologic outcomes (including the results of radioactive iodine scans and postoperative serum thyroglobulin levels) and safety as conventional open procedures (36). Moreover, the robotic approach yielded better outcomes in quality of life and cosmesis (36).

Regarding the 1863 patients with thyroid cancer who underwent total thyroidectomy, therapeutic adjuvant radioactive iodine therapy was performed in 1460 (78.3%). Among these patients, diagnostic whole-body scans showed no abnormal uptake in 1380 patients (94.5%). Furthermore, the serum TSH-suppressed thyroglobulin level was less than 1 ng/mL in 1038 patients (55.7%) at 3 months after surgery. During the follow-up, tumor recurrence was detected by imaging and confirmed in 26 patients (0.5%) (25).

In 2018 we published our initial experience with 250 patients who underwent RATT (37). The final histologic examination reported carcinoma in 103 patients, with a mean diameter of 12.9 mm. According to the European consensus for thyroid cancer management, 26 patients were treated with low radioiodine (I-131) activities (1.1 GBq/30 mCi) for postsurgical thyroid remnant ablation. After 4 years of follow-up, all patients with a thyroid cancer diagnosis were free of disease, and those who underwent total thyroidectomy showed a mean value of 0.8 ± 1.4 ng/mL of TSH-suppressed serum thyroglobulin (37).

As reported in the studies published by the Korean group, we also progressively extended the indications of RATT with the increasing experience, especially for benign lesions. Nevertheless, a careful selection of patients is of paramount importance to achieve excellent results with this technique. To date, we still exclude patients with suspicious VIth level lymph nodes or T4 tumors (37).

Garstka et al. (38) published a comparative study including DTC patients who underwent robot-assisted transaxillary or conventional cervical approach with or without lymph node dissection. A total of 144 surgeries were included, 35 out of 144 were robotics. The Authors reported comparable outcomes in terms of mean tumor size, number of positive microscopic margins and number of lymph nodes removed when lymph node dissections were associated. No statistically significant difference in postoperative thyroglobulin levels was documented, with a comparable follow-up period, and no significant difference in recurrence rate was reported; in particular, no recurrence was reported in the robotic group (38).

Similarly, Noureldine et al. (39) reported their experience with a North America population of patients with thyroid cancer (39). In their study, 35 patients underwent thyroidectomy by means of conventional approach, whereas 25 patients by means of robot-assisted transaxillary approach. They reported that no patient presented high uptake with post-operative I-131 whole body scan and that the mean serum thyroglobulin levels between the two groups were comparable. Moreover, the neck ultrasonography performed on all patients 1 to 3 months after the operation did not show any residual thyroid tissue or evidence of residual or recurrent disease. Although at the 2-year follow-up one patient in the robotic group required reoperation for recurrent disease in the central compartment, the authors concluded that excellent oncologic results can be achieved with RATT in selected patients affected by thyroid cancer (39).

Overall, RATT is feasible and oncologically safe in properly selected patients, and by avoiding a visible neck scar it is associated with excellent cosmesis. This approach proved safe even when applied to patients in the West, whose anthropometric parameters may vary considerably from the Asian population. We believe that in skilled hands, RATT can be considered a valid alternative to CT even in selected patients with thyroid cancer, especially those who have concerns about cosmetic outcome.

We hope that the advent of new medical device companies in the robotic surgical field, the development of new technologies, and the worldwide spread of the technique will gradually break down some limitations of the robotic system, such as the lack of haptic feedback, the long operative time, and the elevated costs, which, however, might be significantly reduced when the procedure is performed by experienced teams and through a limitation of disposable instruments (30, 40). It is worth to underline that at our institution we perform RATT using only 3 robotic arms, with the fourth kept folded: this reduces the length of the incision and the encumbrance of the instruments. Besides, this technique results in lowering the docking time and indirectly the robotic costs, which are further decreased by avoiding the use of the fourth arm. This is especially true with the use of da Vinci Si surgical system, with which the drape for the fourth arm is avoided, differently from the Xi version.

Finally, concerning the economic impact of the robotic procedure, it is important to take into consideration that RATT usually takes a shorter time compared with other robotic operations. This leads to the opportunity of covering empty spaces among the daily operating list and allows an improvement in the efficiency of the robotic operating room.

## Transoral Endoscopic Thyroidectomy With Vestibular Approach (TOETVA)

The only technique that allows a scarless thyroidectomy is the transoral endoscopic thyroidectomy (TOET). Although various techniques for TOET are described, the most used is the TOET with vestibular approach (TOETVA) due to its surgical outcomes and low complication rate (41).

The first attempt of TOET was performed by Witzel et al. (42) using the sublingual route. Many other attempts were performed later on, but all of them were associated with a high complications rate. As result, TOET *via* the sublingual route is no longer performed in clinical practice (41). On the other hand, the first TOETVA was described by Richmon et al. (43). Since then, Anuwong and colleagues (41) refined the technique and performed more than 800 procedures in 2019.

This new natural orifice transluminal endoscopic surgery (NOTES) is performed by means of 3 small incisions (one on the midline for a 5- to 10-mm port and two laterals for 3- to 5-mm ports) in the lower lip’s vestibule, resulting in a truly scarless thyroidectomy. The pre-mandibular space is first created with the help of the dilatator and followed by dissection under direct vision and CO_2_ insufflation (44). Different from other endoscopic thyroid surgery techniques, TOETVA allows an excellent view of the surgical field and equal access to both sides of the central neck; nevertheless, the identification and dissection free of the recurrent laryngeal nerve is approached from top to bottom and may jeopardize the recurrent laryngeal nerve, which usually divides into several branches and therefore must be followed bottom up and not in the opposite direction (45).

Anuwong et al. reported that the eligibility criteria for TOETVA are the following: thyroid gland of a diameter not exceeding 10 cm, comprising either benign thyroid nodule, papillary microcarcinoma with no evidence of metastasis, follicular neoplasm, or well-controlled Graves’ disease. Moreover, they reported that TOETVA can be done safely in patients who had previously undergone surgery or radiation at the chin and neck area (46).

Taking into consideration the limitations of TOETVA and the natural history of differentiated thyroid cancer, Wu et al. (47) reported that this natural orifice transluminal endoscopic approach can be safely performed in case of low-risk thyroid carcinoma up to 2 cm in diameter with adequate oncologic outcomes. Similarly, Anuwong et al. (48) did not consider patients with thyroid malignant tumors larger than 2 cm candidates for TOETVA because it is crucial to extract the tumor intact, and it cannot be morselized as is done with benign nodules.

Chai et al. (49) published in 2017 a retrospective study of 10 female patients who had undergone TOETVA due to papillary thyroid microcarcinoma. Only partial thyroidectomies were included (7 lobectomies and 3 isthmusectomies). The authors documented recurrent laryngeal nerve palsy in 2 patients, fully recovered in 3 months, and acceptable operative times. No oncologic follow-up data were reported (49).

In 2019, Luna-Ortiz et al. (50) performed a retrospective study of 46 patients with DTC who underwent TOETVA, reporting acceptable results. All patients were evaluated for postoperative serum thyroglobulin levels and anti-thyroglobulin antibodies at 4 weeks after surgery, and all exhibited values below 5 ng/dL. The authors claimed that TOETVA may be indicated in case of thyroid carcinoma that is not locally invasive and without lymph nodes involvement (50).

With regard of complications, some studies reported comparable outcomes to CT (46, 51), claiming that TOETVA can be safety performed in selected patients. Anyway, further studies will assess in the future the actual safety of the procedure and the real incidence of some new unconventional complications, such as mental nerve injury, flap perforation and bruising, which are to date rarely reported (46).

In summary, TOETVA is a new technique that provides the best cosmetic results considering that is totally scarless, with a short distance between the thyroid gland and the incisions. Although there are some reports of its feasibility in both benign and malignant lesions of the thyroid, long-term oncologic outcomes regarding thyroid cancer are still lacking. In our opinion, some concerns about this approach still persist, especially regarding the oncologic completeness and the technical feasibility. Besides, Anuwong himself stated that this approach should be used only for 1 to 2 cm thyroid carcinomas (44).

Anyway, since encouraging studies are reported in literature, larger case series with longer follow-up are required to understand the actual oncologic validity and safety of this approach.

## Conclusions

The technological progress has led to the development of several alternative surgical techniques to the thyroid gland, either with a cervical or a remote-access approach, both robotic and endoscopic. Although most of them have been abandoned due to scarcity of quality outcomes, MIVAT and RATT have obtained the consensus from the scientific community. Nowadays, the feasibility and safety of MIVAT has gained widespread acceptance for both benign and malignant diseases, although a minority of patients are eligible for the technique.

Similarly, with the introduction of robotic systems in the surgical armamentarium, RATT progressively became more and more popular among surgeons, initially in Asia and successively in the West, with extension of the indications as the experience increased. To date, thyroid cancer is safely treated by means of RATT, and some case series reported appealing results even when central compartment and lateral neck compartment dissection are associated.

Finally, the recent introduction of TOETVA deserves a special mention because this is the only totally scarless technique to manage thyroid diseases. Notwithstanding, some technical and oncologic concerns about this approach persist, and further research with an adequate follow-up are mandatory to assess its safety, especially in case of malignancy where the integrity of the nodule is essential.

We believe that an accurate patient selection is of paramount importance when a nonconventional approach to the thyroid gland is planned. We strongly encourage that these procedures be centralized to high-volume centers with skilled endocrine endoscopic surgeons.

## Author Contributions

LR wrote the manuscript with the support of PM and GM and analyzed data collected from the literature. Moreover, PM and GM supervised the paper. SB reviewed the final version of the manuscript. All authors contributed to the article and approved the submitted version.

## Conflict of Interest

The authors declare that the research was conducted in the absence of any commercial or financial relationships that could be construed as a potential conflict of interest.
